# Donut Sign on Magnetic Resonance Angiography: Interpret with Caution

**DOI:** 10.5811/cpcem.2018.7.39135

**Published:** 2018-08-15

**Authors:** Kiran K. Gudivada

**Affiliations:** St. John’s Medical College Hospital, Department of Critical Care Medicine, Bangalore, Karnataka, India

## CASE PRESENTATION

We present two cases of cerebrovascular accidents. Case #1: A 24-year-old man presented with open fractures of the left femur and tibia after a motor vehicle collision. Within two hours, he developed left facio-bracial paresis. Although he arrived in the window period for thrombolysis, polytrauma precluded thrombolysis. His modified Rankin Scale (mRS) score at admission was five. Case #2: A 26-year-old man presented to the emergency department after eight hours with hemiplegia and global aphasia. His admission mRS score was four. Stroke workuprevealed hyperhomocysteinemia (>114 μmol/L).

## DIAGNOSIS

In Case #1, considering the background of trauma and imaging suggestive of luminal filling defect, we suspected an internal carotid artery (ICA) dissection.^1^ The proposed mechanism was the hyperextension of the neck due to decelerating forces during trauma, causing stretch of ICA over the cervical vertebrae. This resulted in shear stress and intimal tear, acting as a nidus for the thrombus formation.^2^ In Case #2, hyperhomocysteinemia is the risk factor for thrombus formation leading to stroke. After four weeks, there was a complete resolution of thrombus and the mRS score improved to two in Case #2 with oral antiplatelet and anticoagulation therapy, while Case #1 was lost to follow-up. In both cases, family refused to consent for mechanical thrombectomy.

**Image 1:**
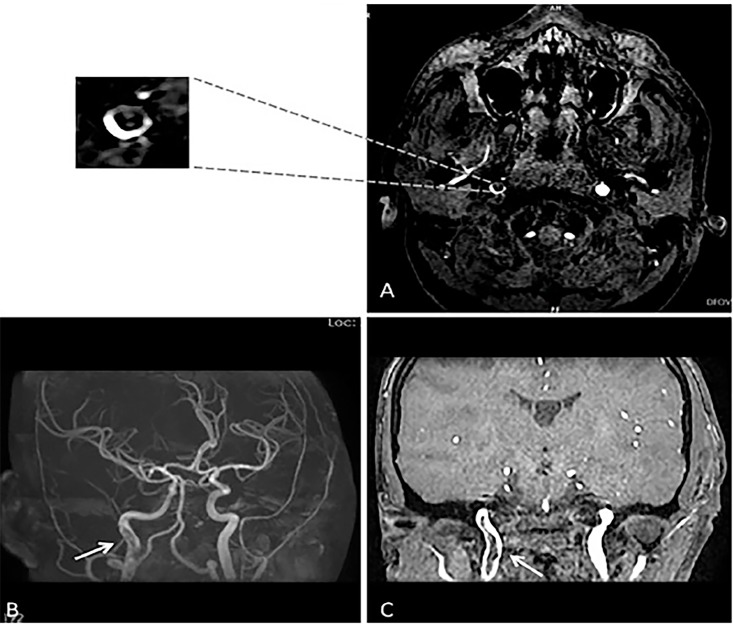
**A)** Axial magnetic resonance angiography (MRA) of the neck shows thrombus in the right internal carotid artery (ICA) (donut sign). **B)** Maximum intensity projection image shows filling defect in the right ICA distal to the bifurcation of common carotid artery (white arrow). **C)** Coronal MRA shows thrombus in the right ICA (white arrow).

**Image 2:**
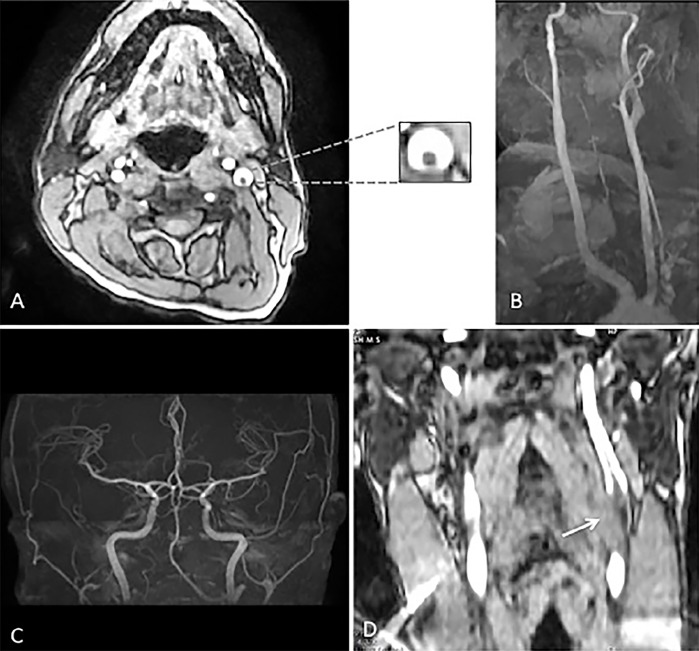
**A)** Axial magnetic resonance angiography (MRA) of the neck shows thrombus in the left internal carotid artery (ICA) (Donut sign), also note an increase in diameter of vessel. **B,C)** Maximum intensity projection images of neck and intracranial vessels shows ‘no definitive filling defect’ in left common carotid artery or ICA. **D)** Coronal MRA shows thrombus in the left ICA (arrow).

**Image 3:**
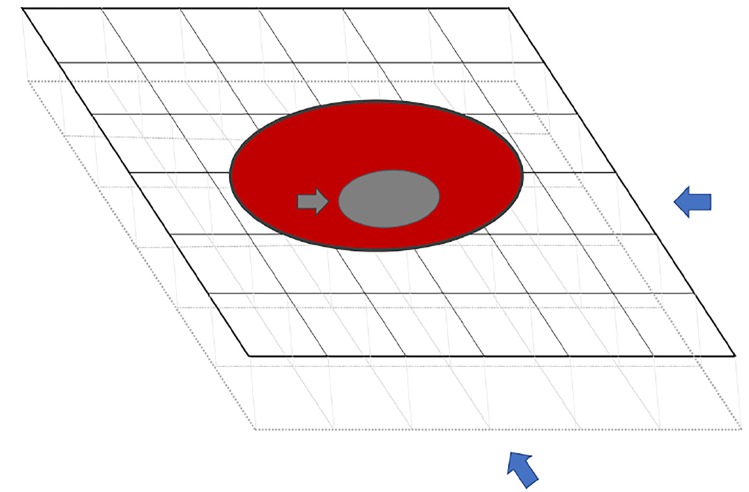
Schematic representation of cross-section of internal carotid artery on voxels. No matter what direction the maximum intensity projection (MIP) reprojection algorithm is oriented (as shown by blue arrows), the hyperintense flowing blood (shown as red) may obscure the intraluminal thrombus (grey arrow) on final MIP.

In the literature, luminal thrombus in the ICA has been described radiologically with various analogies such as “donut sign,” “crescent sign,” or “string sign.”^3^ Although these radiological signs guide us to localize the lesion, they should be interpreted with caution. In Case #1, maximum intensity projection (MIP) image of magnetic resonance angiography (MRA) head showed a filling defect in the right ICA (Image 1B), while in Case #2 there was no definitive filling defect seen in MRA-MIP of the left ICA (Images 2B and 2C). However, thrombus was identified in the axial source images of the neck in both cases (Images 1A and 2A). The probable explanation for the false negative MIP images in Case #2 could be the flow-related alteration in image enhancement. For instance, intraluminal thrombus may become inapparent if surrounded by hyperintense blood flow and a falsely normal-appearing blood vessel can be reconstructed (Image 3).^4^ Therefore, it is always advisable to refer to the individual source images while interpreting the MIP image.^4^

In summary, these images emphasize two practical points: 1) limitations in the interpretation of MRA-MIP image (false negatives); and 2) the significance of evaluating source images of head and neck in stroke workup.

**CPC-EM Capsule**

What do we already know about this clinical entity?

Cerebrovascular accidents are the most common neurological emergencies. Magnetic resonance imaging (MRI) and MR angiography are the recommended modalities to identify stroke etiology.

What is the major impact of the image(s)?

These radiological images highlight the major limitation of maximum intensity projection images in detecting the intraluminal thrombus.

How might this improve emergency medicine practice?

Knowledge of basic MRI interpretation and its limitation will aid the emergency physician in prompt diagnosis, etiological differentiation and subsequent management of stroke.

Documented patient informed consent and/or Institutional Review Board approval has been obtained and filed for publication of this case report.
